# Modern Era in Personalized Medicine of Dual Antiplatelet Therapy After Myocardial Revascularization

**DOI:** 10.3390/jcm15134870

**Published:** 2026-06-23

**Authors:** Amin Dehghan, Niloufar Javadi, Suhail Q. Allaqaband, M. Fuad Jan

**Affiliations:** 1 Department of Surgery, Washington University in St. Louis, St. Louis, MO 63110, USA; damin@wustl.edu; 2 Aurora Cardiovascular and Thoracic Services, Aurora Sinai/Aurora St. Luke’s Medical Centers, Aurora Health Care, Milwaukee, WI 53215, USA; 3 Department of Cardiovascular Medicine, Atrium Health Wake Forest Baptist Medical Center, Winston-Salem, NC 27157, USA

**Keywords:** coronary artery bypass graft, CYP2C19, dual antiplatelet therapy, escalation strategy, integrated risk assessment, myocardial revascularization, platelet reactivity testing

## Abstract

Dual antiplatelet therapy (DAPT) with aspirin and a P2Y12 inhibitor remains the cornerstone of antithrombotic management after myocardial revascularization. However, the traditional “one-size-fits-all” approach to DAPT duration and intensity fails to account for marked interindividual variability in drug response—driven by genetic polymorphisms, notably *CYP2C19* variants like CYP2C19*2, which reach a frequency of up to 75% in specific groups like the Melanesian population—comorbidities such as diabetes and chronic kidney disease, and dynamic clinical factors including age and concomitant medications. We examine the current landscape of precision medicine tools for individualizing DAPT, including platelet function testing, point-of-care genotyping, validated clinical risk scores, and emerging artificial intelligence (AI)–based predictive models. Evidence from landmark trials is synthesized to evaluate escalation, de-escalation, and duration-tailoring strategies within the ischemic–bleeding trade-off framework. Special populations requiring individualized approaches are reviewed, including patients with atrial fibrillation, the elderly, and those requiring urgent noncardiac surgery with perioperative bridging. Future directions, including multi-omics integration, novel antiplatelet agents, and AI-driven clinical decision support systems, are also explored. As a narrative review, conclusions should be interpreted as reflective of current evidence synthesis rather than systematic-review-grade evidence, given the absence of formal risk-of-bias scoring or meta-analytic pooling. Personalized DAPT guided by complementary genetic and phenotypic testing, integrated with dynamic risk stratification, offers a paradigm shift from empiric therapy toward precision-guided antithrombotic management with the potential to simultaneously reduce ischemic and bleeding complications.

## 1. Introduction

Dual antiplatelet therapy (DAPT) consisting of aspirin and a P2Y12 inhibitor has traditionally been the cornerstone of treatment to decrease atherothrombotic risk in patients with acute coronary syndrome (ACS) and stable coronary artery disease (CAD) who undergo a coronary artery bypass graft (CABG) or percutaneous coronary intervention (PCI) with a drug-eluting stent (DES) [1]. Traditionally, guidelines have recommended a standard 12-month duration of DAPT as a “one-size-fits-all” approach after DES placement [2]. However, with the evolution of stent technology and the recognition of the “bleeding-ischemia risk continuum,” a more flexible approach to DAPT duration has emerged. This includes the assessment of the benefits of shorter DAPT duration, ranging from one to three months, with the use of a single antiplatelet agent to minimize the risk of bleeding without compromising ischemic protection [3].

The limitations of standardized DAPT are also reflected in the marked interindividual variability in response to these drugs [1]. Although patients who are classified as “hypo-responders” with high on-treatment platelet reactivity are at significant risk of thrombotic events such as stent thrombosis, “hyper-responders” with low on-treatment platelet reactivity are at risk of bleeding complications [2]. Risk factors that co-exist with these are age, co-morbid conditions such as diabetes and renal failure, and genetic factors, the most important being CYP2C19 loss-of-function (LoF) mutations, which have been reported in Middle Eastern populations, affecting the metabolism of clopidogrel in about 23% of individuals based on genetic testing [1]. Personalized medicine is an effort to rise above the limitations of traditional clinical medicine to achieve the “therapeutic sweet spot” to balance the risks in an individual patient profile [3].

The question that this review sets out to answer is: What is the optimal method of individualization of DAPT in contemporary clinical practice? The authors will discuss the current status of precision medicine tools, including bedside platelet function testing and quick genotyping, and emerging artificial intelligence (AI)-based predictive tools that aim to individually tailor DAPT time and intensity.

## 2. Methodology

**Figure 1:**
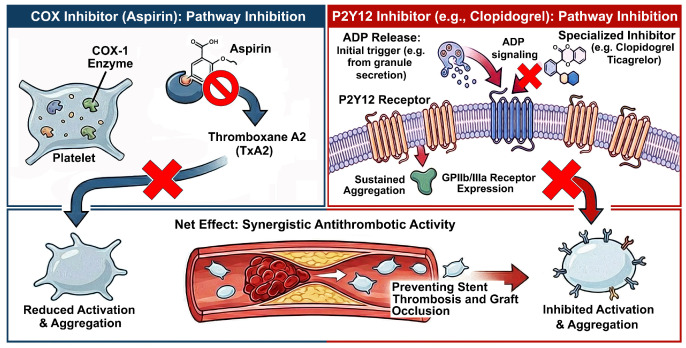
Complementary mechanisms of DAPT. Aspirin inhibits the COX-1 enzyme within platelets, blocking the synthesis of thromboxane A2 (TxA2) and thereby reducing platelet activation and aggregation. Simultaneously, P2Y12 inhibitors (such as clopidogrel or ticagrelor) block ADP signaling at the P2Y12 receptor on the platelet surface, preventing downstream GPIIb/IIIa receptor expression and suppressing sustained aggregation *(Artificial intelligence program Gemini was used in the creation of this graphic, with human oversight and review)*.

**Figure 2:**
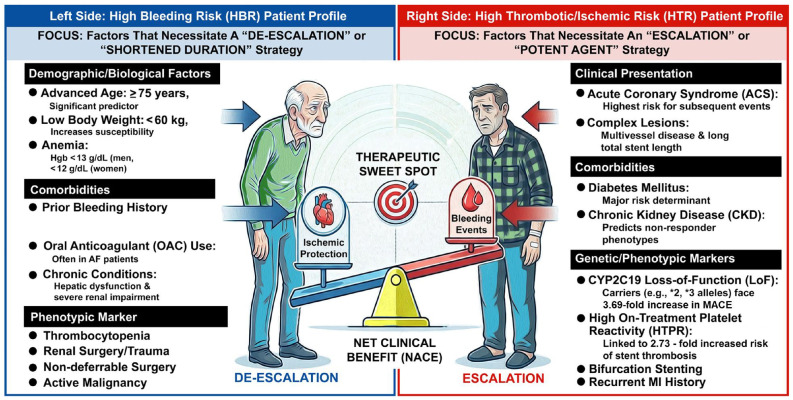
Clinical phenotypes of the ischemic–bleeding trade-off in dual antiplatelet therapy. High bleeding risks/de-escalation: BLEEDSS (bleeding history, low body weight, elderly (≥75 y), existing anemia, drugs (oral anticoagulants), surgery/severe comorbidities (active malignancy, thrombocytopenia, liver or renal disease). High thrombotic risks/escalation: CCLOTSS (CKD and *CYP2C19* LoF, lesion complexity (complex PCI, multivessel disease, long stent length), on-treatment platelet reactivity high (HTPR), type I ACS, sugar (diabetes mellitus), and stent thrombosis) *(Artificial intelligence program Gemini was used in the creation of this graphic, with human oversight and review)*.

This is a narrative review. We searched PubMed/MEDLINE, Embase, Web of Science, and the Cochrane Library for English-language articles published from 2000 to 2026 using combinations of the terms dual antiplatelet therapy, aspirin, clopidogrel, prasugrel, ticagrelor, cangrelor, CYP2C19, platelet function testing, genotyping, de-escalation, DAPT duration, bleeding risk, perioperative management, and coronary guidelines. Randomized trials, meta-analyses, major society guidelines, consensus statements, and high-quality observational studies relevant to individualized antiplatelet therapy after PCI were prioritized. Studies were selected for clinical relevance to the review question rather than by formal systematic review screening, and duplicate, off-topic, or clearly noncomparable reports were excluded. Because this is a narrative review, neither risk-of-bias scoring nor meta-analytic pooling was performed. Figure 1 and Figure 2 were created with the assistance of the Gemini 3.5 Flash tool (Google) under human oversight and editorial review; all figure content was verified by the authors.

## 3. Pharmacological Basis of DAPT

The combination of aspirin and a P2Y12 inhibitor produces synergistic antithrombotic activity because each drug acts on different pathways of platelet activation. The foundation for DAPT is aspirin, which inhibits cyclooxygenase-1 (COX-1) irreversibly, thus inhibiting thromboxane A2 production, a powerful platelet activator [2,4]. In contrast, P2Y12 inhibitors, such as oral drugs clopidogrel, prasugrel, and ticagrelor, and intravenous cangrelor, inhibit adenosine diphosphate (ADP) receptors on the platelet membrane [3,4]. Inhibiting this receptor blocks the final common pathway for platelet activation and subsequent glycoprotein IIb/IIIa receptor expression (Figure 1) [5]. There is large variability in patient response to DAPT drugs, especially clopidogrel, a prodrug that requires two-step hepatic biotransformation [1]. Up to one-third of patients taking clopidogrel develop high on-treatment platelet reactivity, often due to *CYP2C19* LoF variants, significantly increasing the risk of stent thrombosis [6]. In contrast, more potent drugs like prasugrel and ticagrelor have faster and more consistent platelet inhibition [3]. While effective in preventing ischemic events, they have a different efficacy-bleeding risk profile, where increased potency is intrinsically linked to increased risk of major bleeding events [1,3]. DAPT is indicated for a range of clinical conditions. It is the standard of care for ACS/PCI to prevent stent-related and systemic thrombotic events, following CABG to maintain graft patency and reduce long-term mortality, and for chronic coronary syndrome following stent implantation [1,7,8,9]. Beyond the coronary arteries, DAPT is indicated for secondary prevention in patients with minor ischemic stroke or transient ischemic attack to reduce early recurrence, and for managing peripheral artery disease and carotid disease [10]. Beyond these established coronary and cerebrovascular indications, DAPT or antiplatelet combination therapy is also employed following several structural heart interventions. Following transcatheter aortic valve implantation, short-duration DAPT (1–3 months) was historically recommended to reduce leaflet thrombosis and embolic risk [11]; however, current evidence from the Antiplatelet Therapy for Patients Undergoing Transcatheter Aortic Valve Implantation (POPular-TAVI) trial has shifted practice toward aspirin monotherapy in anticoagulant-naive patients, given a superior bleeding safety profile without a meaningful difference in ischemic outcomes [12]. Following left atrial appendage occlusion with the Watchman device (Boston Scientific, Marlborough, MA, USA), DAPT for 45 days followed by clopidogrel monotherapy through 6 months is standard protocol to minimize device-related thrombus while the endothelialization process completes [13]. Transcatheter mitral valve interventions and patent foramen ovale closure procedures similarly mandate short-term antiplatelet regimens, though evidence for device-specific optimal durations remains limited [14].

## 4. Why Personalization? The Ischemic–Bleeding Trade-Off

### 4.1. Ischemic Risk Determinants

Ischemic risk is mainly determined by clinical presentation, with patients presenting with ACS or prior myocardial infarction (MI) at highest risk for subsequent events [3]. Diabetes mellitus and chronic kidney disease (CKD) are well-established major risk determinants that significantly elevate thrombotic risk and alter drug responsiveness, as recognized by the 2024 International Consensus Statement [15] and ESC guidelines [16,17]. Genetic factors, including loss-of-function mutations in *CYP2C19*, significantly elevate major adverse cardiovascular event (MACE) risk (HR = 3.69, 95% CI 1.69–8.05) [18]. Also, ischemic risk is increased in patients with the CYP2C19*2 variants by 18–25% [1,19]. Additionally, high on-treatment platelet reactivity (HTPR) is strongly associated with stent thrombosis (RR 2.73, 95% CI 2.03–3.69) and non-fatal MI (HR: 3.36) [15,20]. Procedural factors like lesion complexity, multivessel disease, and total stent length further modulate this risk profile (Figure 2) [3,19].

### 4.2. Bleeding Risk Determinants

One of the most important factors for bleeding risk is advanced age, followed by a history of prior bleeding events [3,15]. Biological factors include anemia and systemic impairments such as hepatic dysfunction [10]. The use of oral anticoagulants significantly increases bleeding risk, often requiring less intensive antiplatelet regimens [3]. Patients experiencing low platelet reactivity are at a 1.74-fold increased risk for bleeding events without experiencing additional ischemic protection [15]. Hypertension, low body weight, and other medications used are other factors to be considered in patient-specific risk models (Figure 2) [2,10].

### 4.3. Quantifying Risk: Clinical Scores and Consensus Definitions

Clinical risk scores (Table 1) are the basis for the scientific approach in managing the trade-off between ischemia and bleeding. For instance, the DAPT score, based on 11,648 patients, uses five clinical and three procedural parameters for the identification of extended DAPT candidates [21]. In contrast, the PRECISE-DAPT risk score uses five clinical predictors, including age, creatinine clearance, hemoglobin, white blood cell count, and spontaneous bleeding, where a PRECISE-DAPT risk score ≥ 25 identifies high bleeding risk (HBR) patients [22]. Similarly, the ARC-HBR criteria offer a semiquantitative approach for defining high risk of bleeding, where 13 clinical parameters identify HBR, including one major and two minor criteria [23]. In contrast, the PARIS score uses six clinical parameters for thrombosis and bleeding risk, with c-indices of 0.65 and 0.64, respectively [24]. Thus, scientific evidence from clinical trials is challenging the static approach of the DAPT recommendations, thus emphasizing the need for individualized approaches rather than blanket DAPT recommendations. These scores should be interpreted alongside contemporary European guideline documents, particularly the 2023 European Society of Cardiology (ESC) ACS guideline [16] and the 2024 ESC chronic coronary syndromes guideline [17], which explicitly incorporate risk-adapted antiplatelet strategies rather than a uniform DAPT duration.

### 4.4. Integrated Clinical-Genetic Scores

A study developed a score called the ABCD-GENE score (Age, BMI, CKD, Diabetes, and Genotyping) to increase accuracy by using a scoring system based on biological and genetic markers. This score assigns weighted points (e.g., +24 for two *CYP2C19* loss-of-function (LoF) alleles, +4 for age > 75) to predict clopidogrel response. Patients with an ABCD-GENE score > 10 are associated with a 48% increase in MACE and a 54% increase in death [25].

### 4.5. Net Clinical Benefit and Decision-Making Limits

The concept of net adverse clinical events (NACE) has been developed to offer a framework for clinical decision-making by striking a balance between the occurrence of ischemic and bleeding events [26,27]. Since the risks of thrombotic and bleeding events coexist, the therapeutic window must be optimized to reach the best possible results in the context of patient safety [1,15]. Contemporary AI-based models have been reported to improve net clinical outcomes relative to fixed-duration approaches; however, these quantitative claims (e.g., 31% reduction in bleeding events) originate solely from a single, non-peer-reviewed preprint [19] and require independent validation before clinical adoption.

Structured assessments should be used alongside clinical judgment, as traditional models often show only moderate ability to distinguish risk (c-statistics: 0.65–0.75), which is why guideline committees give them a Class IIb recommendation [3]. Modern AI-driven models have shown promising but preliminary improvements over traditional risk scores. One non-peer-reviewed preprint reported superior predictive performance compared with traditional models; however, these findings have not been externally validated, and the model has not been prospectively tested in a randomized trial [19].

## 5. Pharmacogenomics and Precision Antiplatelet Therapy

### 5.1. CYP2C19 Genetic Variability

The *CYP2C19* gene encodes the hepatic enzyme responsible for converting the prodrug clopidogrel into its active metabolite, and its genetic variability is the key cause of non-responsiveness [1,15]. The most common LoF alleles are CYP2C19*2 and CYP2C19*3, which produce dysfunctional enzymes, thereby reducing the production of the active metabolite [5]. CYP2C19*2 accounts for over 95% of LoF status in Black and White populations and 75% in Asian populations, i.e., Melanesian populations, in which the prevalence of CYP2C19*2 can reach 75% [5,15,28]. On the other hand, CYP2C19*17 is a gain-of-function allele, which codes for increased transcription and ultra-rapid metabolism [3].

### 5.2. Clinical Impact on Clopidogrel Response

Individuals who have LoF alleles have an increased risk of HTPR and adverse cardiovascular outcomes. Indeed, LoF individuals are associated with increased risk of MACE compared with non-carriers (HR= 3.69, 95% CI 1.69–8.05) [18]. In the TRITON-TIMI 38 (TRial to assess Improvement in Therapeutic Outcomes by optimizing platelet inhibitioN with prasugrel Thrombolysis In Myocardial Infarction 38) study, LoF carriers had a significantly higher primary outcome rate (12.1% vs. 8.0%, *p* = 0.01), while homozygous carriers faced a 3.97-fold higher risk of stent thrombosis [5,29]. Notably, the GRAVITAS (Gauging Responsiveness with A VerifyNow assay—Impact on Thrombosis And Safety) trial demonstrated that simply increasing the clopidogrel dose in LoF-associated hypo-responders is insufficient to overcome this genetic disadvantage [30].

### 5.3. Point-of-Care Genotyping and Clinical Implementation

Point-of-care genotyping using the Cube System (Genomadix) or a gene drive enables immediate antiplatelet selection by providing rapid results from buccal swab samples within less than 1 h [15]. The QPGx-CARES (Qatar Pharmacogenetics Clinical Applications and Research Enhancement Strategies) program showed the efficacy of this approach by identifying actionable LoF alleles in 22.9% of patients in Middle Eastern populations, prompting a switch in therapy to appropriate agents such as ticagrelor [1]. This approach has the advantage of 80% adoption and 100% adherence for poor metabolizers.

### 5.4. Guideline and Regulatory Perspectives

The Clinical Pharmacogenetics Implementation Consortium (CPIC) 2022 update and Dutch Pharmacogenetics Working Group (DPWG) guidelines recommend using alternative P2Y12 inhibitors (prasugrel or ticagrelor) in patients with confirmed LoF alleles [31,32]. Further, recent scientific statements from the American Heart Association (AHA) emphasize the importance of routine genotyping in the selection of antiplatelet agents in PCI practice [33]. The 2023 ESC ACS guidelines support genotype-guided de-escalation in selected patients (Class IIb), and the 2024 ESC CCS guidelines assign clopidogrel monotherapy after completion of DAPT a Class I, level of evidence A recommendation. Accordingly, the absence of a genotype-based recommendation in the 2021 American College of Cardiology (ACC)/AHA/Society for Cardiovascular Angiography and Interventions (SCAI) guidelines for coronary artery revascularization and the 2023 ACC/AHA chronic coronary disease guidelines should not be interpreted as evidence against genotype-guided care. Pharmacogenetic guidelines (CPIC, DPWG, AHA Scientific Statement) are developed by pharmacology and pharmacogenomics consortia whose threshold for recommendation is evidence of predictable drug–gene interactions, not necessarily cardiovascular outcome benefit demonstrated in prospective randomized trials. By contrast, ACC/AHA cardiology guidelines require randomized controlled trial outcome data before issuing Class I or IIa recommendations; currently, no large, prospective randomized controlled trial has demonstrated statistically significant reductions in MACE with genotype-guided DAPT selection as a primary endpoint, explaining the Class IIb or absent recommendation in ACC/AHA documents. The AHA 2024 Scientific Statement supports *CYP2C19* testing at a level equivalent to Class IIb—it is reasonable and may benefit patients, but is not yet mandated. Clinicians should understand this distinction: absence of a strong ACC/AHA guideline recommendation reflects an evidence gap in prospective trials, not a safety concern or active evidence against efficacy. This distinction underscores why the recommendation strength differs between pharmacogenetic bodies (strong recommendation based on drug–gene evidence) and cardiology guidelines (Class IIb based on absence of outcome trial data). Also, the 2024 International Expert Consensus Statement supports the selective use of genetic testing, particularly for DAPT de-escalation when bleeding risk exceeds thrombotic risk and for DAPT escalation when thrombotic risk exceeds bleeding risk [15].

## 6. Platelet Function Testing

### 6.1. Methods and Clinical Relevance

Platelet function testing (PFT) provides a direct, phenotypic measure of an individual’s response to antiplatelet agents. There are several methods to determine it; however, light transmittance aggregometry is the historical gold standard, defining HTPR at a maximal aggregation rate > 46% (5 mM ADP) or >59% (20 mM ADP) [4,34]. Due to the complexity of light transmittance aggregometry, point-of-care assays are preferred for bedside use; the VerifyNow P2Y12 assay (Werfen, Bedford, MA, USA) identifies HTPR at >208 platelet reactivity units (PRU), while the Multiplate analyzer (Roche Diagnostics, IN, Indiana, USA) uses a cutoff of >46 U [35]. Additionally, the AGGRESTAR PL-12 system (SINNOWA Medical Science & Technology Co., Nanjing, Jiangsu Province, China) utilizes a platelet count drop method to define HTPR as a maximum aggregation rate > 55%, showing high consistency with VerifyNow (r = 0.829) [27,36]. The 2019 expert consensus by Sibbing et al. further standardized PFT recommendations specifically for guiding P2Y12 inhibitor selection after PCI, endorsing the VerifyNow PRU assay as the preferred point-of-care platform and formalizing the PRU-based therapeutic window (85–208 PRU) as a clinically actionable, evidence-integrated target that incorporates both ischemic and hemorrhagic risk thresholds into a single actionable decision framework [37].

### 6.2. High and Low Platelet Reactivity

The clinical application of PFT is its predictive power for adverse events. Patients with HTPR face a 2.73-fold increased risk of stent thrombosis (RR: 2.73; 95% CI: 2.03–3.69) and a 3.36-fold higher risk of nonfatal MI [15,20]. In contrast, low platelet reactivity is associated with a 1.74-fold higher risk of major bleeding [38]. Being in the therapeutic window (e.g., 85–208 PRU) is considered an independent predictor for a 53% reduction in NACE (OR: 0.47) [2,20].

### 6.3. Complementarity with Genetic Testing

PFT and genetic testing are synergistic for personalization. While genetic testing identifies a patient’s fixed metabolic condition (e.g., *CYP2C19* status), it only explains approximately 15% of the variability in clopidogrel response [39]. PFT gives us the influence of dynamic factors such as age, diabetes, and drug–drug interactions [15]. Applying both ensures that “hypo-responders” with LoF genotypes are effectively escalated to potent agents, while “hyper-responders” are identified for safe de-escalation to decrease the chance of bleeding. The 2019 expert consensus by Sibbing et al. explicitly endorsed a stepwise integrative approach: initial *CYP2C19* genotyping to rapidly identify LoF allele carriers, followed by confirmatory PFT in patients with borderline or discordant genetic results [37].

## 7. Personalizing DAPT Intensity: Escalation and De-Escalation

### 7.1. Escalation Strategies

The antiplatelet escalation strategy targets the reduction of ischemic events by intensifying the platelet inhibition process through switching patients from clopidogrel to more effective P2Y12 inhibitors, including prasugrel or ticagrelor [3]. Such an escalation is essential in patients with clopidogrel resistance proven by PFT or genotyping [15]. The PATH-PCI (Personalized Antithrombotic Therapy for Coronary Heart Disease after PCI) study found a 32% decrease in NACE rate among chronic coronary syndrome patients receiving PFT-based antiplatelet management (HR: 0.68; 95% CI: 0.49–0.95) [40]. Furthermore, the PHARMCLO (Pharmacogenetics of Clopidogrel in Patients With Acute Coronary Syndromes) study reported a 42% NACE reduction rate due to genotype-based antiplatelet escalation (HR: 0.58) [41]. These findings are consistent with the Class I, Level A recommendation issued in the 2017 ESC focused update on DAPT by Valgimigli et al., which endorsed escalation from clopidogrel to ticagrelor or prasugrel as standard of care in ACS patients undergoing PCI in the absence of contraindications such as prior intracranial hemorrhage, active pathological bleeding, or concomitant oral anticoagulation [42]. The 2018 ESC/EACTS guidelines on myocardial revascularization reinforced this hierarchy, explicitly stating that ticagrelor 90 mg twice daily or prasugrel 10 mg once daily should be preferred over clopidogrel in the ACS-PCI context, with clopidogrel reserved for patients in whom the more potent agents are contraindicated or cost-prohibitive [43].

### 7.2. De-Escalation Strategies

The de-escalation strategy tries to minimize bleeding events while maintaining efficacy. The guided approach employs PFT or genotyping to safely change patients who qualify as high responders to clopidogrel from the use of stronger P2Y12 inhibitors [15]. An example of an unguided approach includes switching patients based on clinical decision, as in the case of TALOS-AMI (TicAgrelor Versus CLOpidogrel in Stabilized Patients With Acute Myocardial Infarction) trial, where there was a significant reduction in NACE rate (HR: 0.55; *p* = 0.0001) through switching patients to clopidogrel at one-month post-PCI [44]. The 2017 ESC focused update by Valgimigli et al. provided a structured, evidence-based framework for such decisions by introducing the PRECISE-DAPT score as a validated bleeding-risk instrument: patients scoring ≥25 points are identified as high-bleeding-risk candidates for abbreviated DAPT (3–6 months), while those below this threshold are advised to complete the standard 12-month course. The guidelines additionally recommended that when de-escalation is performed, it should ideally be guided by PFT or genotyping rather than clinical assessment alone, to ensure that patients being transitioned to clopidogrel retain sufficient on-treatment platelet inhibition [42].

### 7.3. Evidence from Clinical Trials

The TOPIC (Timing of Platelet Inhibition After Acute Coronary Syndrome) trial demonstrated that unguided switching to clopidogrel during the first month after PCI for ACS was associated with a 52% decrease in NACE (HR: 0.48; *p* < 0.01) [45]. The TROPICAL-ACS (Testing Responsiveness To Platelet Inhibition On Chronic Antiplatelet Treatment For Acute Coronary Syndromes) study demonstrated that guided de-escalation using PFT was as effective as conventional prasugrel treatment [46]. In addition, the HOST-REDUCE-POLYTECH-ACS (Harmonizing Optimal Strategy for Treatment of coronary artery diseases-comparison of Reduction of Prasugrel Dose or Polymer Technology in ACS patients) trial confirmed the effectiveness of dose de-escalation, proving that prasugrel dose reductions to 5 mg per day during the first month reduced NACE by 30% (HR: 0.70; *p* = 0.012) [47].

### 7.4. Timing Considerations and Early Phase Risks

Timing plays an important role since the first 30 days post-PCI carry the highest risk for thrombotic events. PFT-guided de-escalation may be constrained by the wash-in phenomenon when the patient is changed to clopidogrel before undergoing a PFT test, putting a low responder at risk of experiencing ischemia during the initial treatment stage [15]. Precision tools facilitate the identification of a therapeutic “sweet spot” at an early stage of treatment [3].

## 8. Antiplatelet Management After CABG

Aspirin remains the established foundation of antithrombotic therapy following CABG, and the benefit of adding a P2Y12 inhibitor continues to be debated, with the existing literature yielding controversial conclusions that underscore the need for individualized decision-making.

A meta-analysis by Agrawal et al., pooling data from 38 trials including more than 77,000 participants, demonstrated reductions in all-cause and cardiovascular mortality with DAPT, an effect predominantly observed among patients who underwent CABG in the setting of ACS, despite increased risk of major and minor bleeding [48]. A separate pooled analysis by Agarwal et al. similarly reported lower graft occlusion rates (RR 0.79) and reduced mortality (RR 0.67) with dual therapy, without a statistically significant increase in major bleeding events [49]. Taken together, this evidence precludes a uniform prescribing approach and reinforces the imperative to weigh individual thrombotic and hemorrhagic risk profiles before initiating DAPT in post-CABG patients.

The choice of P2Y12 agent introduces an additional layer of complexity. The AHA has issued a Class IIa Level B recommendation favoring ticagrelor–aspirin over clopidogrel–aspirin for secondary ischemic prevention after CABG [50], supported by data from the DACAB (Different Antiplatelet Therapy Strategy After Coronary Artery Bypass Graft Surgery) trial demonstrating superior saphenous vein graft patency at one year with ticagrelor-based DAPT compared with aspirin monotherapy [51]. Consistent with this, Wang et al. reported lower mortality and lower rates of MACE with ticagrelor-based regimens versus clopidogrel-based regimens, without a significant difference in bleeding rate [52]. In contrast, European guideline committees have adopted a more conservative posture toward routine DAPT in stable post-CABG patients, citing the POPular-CABG (The Effect of Ticagrelor on Saphenous Vein Graft Patency in Patients Undergoing Coronary Artery Bypass Grafting Surgery) and TARGET (Comparison Three vs. Twelve Months of Dual Anti-Platelet Therapy After Stent Implantation) trials, which failed to demonstrate a patency benefit with more intensive P2Y12 receptor blockade. A review by Bhatt et al. highlights these conflicting findings as illustrative of the unresolved question of optimal DAPT duration in this population [53]. In the absence of a robust consensus, an individualized risk-stratified strategy remains the most defensible clinical approach.

Considering perioperative management in patients undergoing elective CABG, the available evidence supports deferring surgery for approximately three months following prior PCI, with resumption of DAPT within 24 to 48 h postoperatively once hemostasis has been secured [54]. A systematic review demonstrated that brief P2Y12 interruption in patients with drug-eluting stents carries a relatively low risk of stent thrombosis, provided aspirin is maintained throughout the perioperative period; the median time to a thrombotic event under these circumstances exceeds 12 days [55]. Conversely, continuation of P2Y12 inhibitors through the time of CABG has not been shown to improve survival in stable patients and is associated with higher rates of bleeding and transfusion [56], further supporting selective and risk-adapted discontinuation strategies (Table 2).

The 2018 ESC/EACTS guidelines on myocardial revascularization provide important context for the choice between PCI and CABG and the subsequent antithrombotic approach [43]. These guidelines recommend that patients with multivessel CAD, particularly those with complex coronary anatomy (SYNTAX score > 22) or diabetes mellitus, be preferentially directed to CABG by the multidisciplinary heart team, given superior long-term outcomes in such cohorts. From an antiplatelet standpoint, the 2018 guidelines specify that clopidogrel is the preferred P2Y12 inhibitor following CABG when dual antiplatelet therapy is indicated, as neither prasugrel nor ticagrelor has demonstrated a definitive net benefit over clopidogrel in the post-surgical setting, and both agents carry a potentially higher hemorrhagic burden in this context [43].

## 9. Antiplatelet Considerations After Off-Pump CABG

Off-pump CABG presents distinct antiplatelet management challenges relative to conventional on-pump surgery [62]. Meta-analytic data have demonstrated a significantly higher overall graft failure rate following off-pump CABG compared with on-pump CABG (RR 1.35; 95% CI 1.16–1.57), with an even more pronounced effect for venous conduits (RR 1.41; 95% CI 1.24–1.60) [63], providing a pathophysiological rationale for considering more intensive antiplatelet strategies in this population, particularly in recipients of saphenous vein grafts.

An emerging paradigm in off-pump CABG management integrates *CYP2C19* genotyping with PFT to guide individualized DAPT selection. In a retrospective cohort of 1134 consecutive off-pump CABG patients, a personalized strategy employing both *CYP2C19* genotyping and platelet function assessment to guide the choice between clopidogrel- and ticagrelor-based DAPT rather than applying a uniform aspirin-plus-clopidogrel regimen resulted in a significantly lower incidence of MACE compared with the standard DAPT group (5.5 vs. 9.2%; HR 0.583; 95% CI 0.371–0.915; *p* = 0.019), driven primarily by a reduction in MI (1.7 vs. 4.2%; HR 0.407; 95% CI 0.196–0.846; *p* = 0.016), without a significant difference in Thrombolysis in MI (TIMI) major bleeding rates (5.3 vs. 6.0%; RR 0.883; 95% CI 0.537–1.453; *p* = 0.626) [60]. These findings extend the precision antiplatelet paradigm, well-established in PCI populations, to the surgical revascularization setting and support the broader application of genotype- and phenotype-guided antiplatelet selection following off-pump CABG (Table 2).

## 10. Perioperative Management of P2Y12 Inhibitors: Discontinuation Timing and Bridging Across Surgical Settings

Perioperative P2Y12 discontinuation requires careful calibration of surgical bleeding risk against the risk of thrombotic events in the interval without antiplatelet protection. Pooled analyses from contemporary reviews have demonstrated that late P2Y12 discontinuation, defined as fewer than five days before the operative procedure, is associated with a 2.5-fold higher risk of reoperation for bleeding and a 1.5-fold higher risk of mortality compared with earlier cessation [64].

The 2018 ESC/EACTS revascularization guidelines codified discontinuation intervals as Class II recommendations for patients undergoing elective CABG: ticagrelor should be withheld for at least 3 days, clopidogrel for at least 5 days, and prasugrel for at least 7 days, preoperatively. Importantly, the same guidelines endorse point-of-care PFT as a Class IIa tool to guide surgical timing on an individualized basis, recognizing that fixed washout periods may either overestimate or underestimate residual platelet inhibition in individual patients depending on pharmacokinetic variability, patient age, and renal function [43]. The 2017 ESC DAPT update complementarily emphasized that aspirin should be maintained throughout the perioperative period in all patients with prior coronary stenting, underscoring its fundamental role in preventing early graft and stent thrombosis during the P2Y12-free interval [42].

The clinical significance of discontinuation timing is particularly pronounced in patients with a recent PCI. In a cohort of 133 patients undergoing cardiac surgery within one year of PCI, perioperative P2Y12 inhibition per se was not independently associated with higher rates of MACE or bleeding; however, continuation of the agent until the day of surgery conferred a significantly elevated bleeding risk (adjusted OR 2.93; 95% CI 1.53–5.59) [65]. In that same cohort, a PCI-to-surgery interval of ≤30 days was predictive of MACE, while emergent or urgent operative status predicted bleeding, findings that underscore the multidimensional nature of perioperative risk stratification. In a separate observational cohort of 2090 PCI patients, early clopidogrel discontinuation was uncommon; however, among patients in whom both bleeding and drug interruption occurred, the hazard ratio for MACE reached 9.34 (95% CI 3.39–25.70) [66], highlighting the severe ischemic consequences of unplanned antiplatelet interruption in the perioperative window.

Bridging antiplatelet therapy is not routinely indicated but merits consideration in select patients at very high thrombotic risk who cannot safely remain on oral DAPT through the perioperative period. Cangrelor, a rapidly reversible intravenous P2Y12 inhibitor, represents the most thoroughly studied bridging agent in this context. Its ultrashort plasma half-life of approximately 3 to 5 min enables platelet function recovery within 60 min of cessation, thereby confining the perioperative bleeding window to a degree that is unattainable with oral agents [67]. Feasibility and safety data from a real-world multicenter Italian registry enrolling 24 patients with high thrombotic risk undergoing nondeferrable intermediate-to-high-bleeding-risk surgery support the use of a standardized cangrelor bridging protocol at 0.75 μg/kg/min, initiated approximately three days after oral P2Y12 discontinuation and discontinued a mean of 6.6 h before surgical incision, without significant hemorrhagic or thrombotic complications [68]. It must be acknowledged, however, that the current evidence base supports feasibility rather than definitive outcome superiority and remains limited by small sample sizes and registry-level methodology. Accordingly, bridging should be reserved for patients with the highest ischemic risk profiles, particularly those with recent or complex stent implantation, and decisions regarding its use should be made collaboratively among cardiology, anesthesiology, and surgical teams (Table 3).

## 11. Personalizing DAPT Duration After PCI for ACS

### 11.1. From Fixed to Tailored Duration

The concept of antiplatelet therapy is gradually moving away from a fixed course of treatment lasting 12 months and toward risk-adapted treatment strategies where both thrombotic and bleeding risk compete [2,3]. Contemporary evidence from landmark randomized trials demonstrates that both excessively short and excessively prolonged DAPT carry meaningful trade-offs between ischemic and hemorrhagic outcomes, showing the need for individualized, rather than uniform, duration decisions (Table 4) [69,70,71,72]. Static models are increasingly replaced by dynamic assessments that consider clinical presentation and procedural complexity [3].

**Figure 3:**
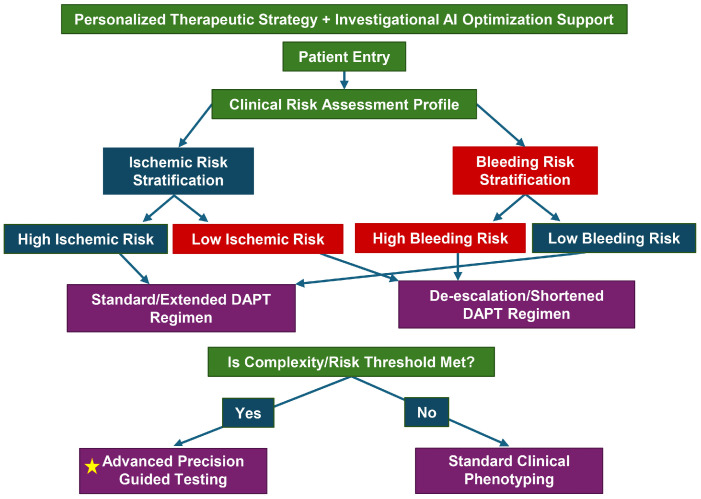
Proposed framework for personalized DAPT after CABG and PCI, integrating clinical risk assessment with selected precision medicine and emerging AI-based approaches. Yellow star: *CYP2C19* Genotyping (Metabolizer Status)/Verify Now/Platelet Function (PRU level).

Figure 3 presents a proposed framework integrating clinical risk scores, *CYP2C19* genotyping, platelet function testing, and emerging AI-based tools for personalized DAPT decision-making after myocardial revascularization.

### 11.2. Evidence from Landmark Trials

The STOPDAPT-2 (Short and Optimal Duration of Dual Antiplatelet Therapy After Everolimus-Eluting Cobalt-Chromium Stent-2) study showed that 1 month of DAPT + clopidogrel was associated with an improved outcome compared with the 12-month regimen, with rates of primary outcomes equal to 2.36% vs. 3.7%, respectively (HR: 0.64; *p* = 0.04) [69]. In a trial that enrolled only patients with high bleeding risk (HBR) (Management of High Bleeding Risk Patients Post Bioresorbable Polymer Coated Stent Implantation With an Abbreviated Versus Prolonged DAPT Regimen: MASTER-DAPT), short-term discontinuation (1 month) of DAPT proved not inferior to standard DAPT (at least 3 months) in NACE and MACE but substantially reduced major bleeding episodes [70]. Moreover, evidence from the SMART-DATE (Smart Angioplasty Research Team: safety of six-month duration of Dual Antiplatelet Therapy after Percutaneous Coronary Intervention in Patients with Acute Coronary Syndromes) and REDUCE ACS (Short-term Dual Anti Platelet Therapy in Patients With ACS Treated With the COMBO Dual-therapy Stent) trials suggests that early stoppage of P2Y12 inhibitor (within 3–6 months) is non-inferior to 12-month DAPT in ACS patients [71,72].

### 11.3. P2Y12 Monotherapy and Aspirin-Free Strategies

The new aspirin-free approaches are based on monotherapy with P2Y12 inhibitor following an ultra-short DAPT period of 1–3 months to increase safety [3]. According to results from the TWILIGHT (Ticagrelor with Aspirin or Alone in High-Risk Patients after Coronary Intervention) study, 3 months of monotherapy with ticagrelor reduced the risk of bleeding by 44% (HR: 0.56; *p* < 0.001) without sacrificing efficacy compared with standard dual treatment [73]. Likewise, the ULTIMATE-DAPT study revealed that the use of monotherapy with ticagrelor after 1 month of DAPT produced a significant decline in major bleeding to 2.1% among ACS patients [74]. These findings are further supported by a systematic review and meta-analysis by Alagna et al., which pooled data from TWILIGHT, ULTIMATE-DAPT, and related trials and confirmed that ticagrelor monotherapy following short-duration DAPT significantly reduces clinically relevant bleeding (RR 0.53; 95% CI 0.37–0.77) without increasing ischemic events [75]. These evolving aspirin-free strategies are situated within the broader landscape described by Navarese et al. in their 2023 network meta-analysis of antithrombotic strategies within and beyond 12 months in established CAD. Pooling data across multiple DAPT duration and composition trials, that analysis identified ticagrelor monotherapy following a short initial DAPT period as the regimen achieving the most favorable balance of ischemic protection and bleeding safety at long-term follow-up, superior to both extended DAPT and aspirin monotherapy for net adverse events. It also confirmed that DAPT prolongation beyond 12 months reduces MI rates at the expense of significantly increased major bleeding, thereby reinforcing individualized rather than uniform prolongation strategies [76].

### 11.4. Patients with High Bleeding Risk

In HBR patients identified using The Academic Research Consortium for High Bleeding Risk (ARC-HBR) criteria or a PRECISE-DAPT (Predicting Bleeding Complications in Patients Undergoing Stent Implantation and Subsequent Dual Antiplatelet Therapy) score > 25, ultra-short DAPT therapy (1–3 months) is recommended [77]. A recent preprint study, not yet peer-reviewed, has suggested that AI-driven stratification may have the potential to reduce overtreatment in low-risk patients and optimize therapy duration in high-risk individuals; however, these findings require prospective validation before informing clinical practice [19].

### 11.5. Clopidogrel Versus Aspirin Monotherapy at Long-Term Follow-Up After PCI

An emerging clinical question following the establishment of short-duration DAPT and P2Y12 monotherapy strategies is whether clopidogrel monotherapy is superior to aspirin monotherapy for long-term maintenance antiplatelet therapy after PCI. Several recent studies have addressed this question. The STOPDAPT-2 ACS (Short and Optimal Duration of Dual Antiplatelet Therapy-2 Study for the Patients With Acute Coronary Syndrome) study on 2986 patients revealed that after 5 years of follow-up, clopidogrel monotherapy was superior to aspirin in reducing cardiovascular events after PCI, with no increase in major bleeding, supporting its long-term use beyond 1 year [78]. The HOST-EXAM (Harmonizing Optimal Strategy for Treatment of Coronary Artery Stenosis–Extended Antiplatelet Monotherapy) extended follow-up data published in 2022 demonstrated that clopidogrel monotherapy was associated with a significantly lower rate of composite ischemic and bleeding endpoints compared with aspirin monotherapy during long-term follow-up after PCI, providing evidence favoring clopidogrel over aspirin as the preferred maintenance agent [79]. This was complemented by a meta-analysis that pooled data from HOST-EXAM, STOPDAPT-2, and related trials, confirming that P2Y12 inhibitor monotherapy with ticagrelor or clopidogrel was associated with fewer net adverse events than aspirin monotherapy in 5.5 years of post-PCI follow-up. Recent data published in *Lancet* (2025) [80], *JAMA Cardiology* (2025) [81], and *Medicine* (2026) [82] further support clopidogrel monotherapy as both effective and cost-efficient in broader contemporary PCI cohorts. Collectively, these studies shift the landscape of long-term post-PCI antiplatelet maintenance therapy toward routine consideration of clopidogrel monotherapy over aspirin in appropriately selected patients who have completed an initial DAPT period.

## 12. Special Populations and Complex Clinical Scenarios

### 12.1. Patients with Atrial Fibrillation Requiring Anticoagulation

The management of patients with atrial fibrillation who require PCI involves switching from triple antithrombotic therapy to dual antithrombotic therapy. Guidelines suggest the use of aspirin only for the peri-procedure period (shorter than 1 week), followed by oral anticoagulants and a P2Y12 inhibitor, preferably clopidogrel, to avoid bleeding [3,15]. While dual antithrombotic therapy considerably decreases the chances of bleeding, a sub-analysis of the AUGUSTUS trial showed that it increased the chances of stent thrombosis more than triple antithrombotic therapy [83]. A recent preprint study, not yet peer-reviewed, has highlighted atrial fibrillation as a variable carrying both ischemic and hemorrhagic complexity in AI-based risk models, illustrating the direction this field is heading [19]. The 2017 ESC DAPT update directly addressed this complex population by recommending clopidogrel as the preferred P2Y12 agent when oral anticoagulation is required in AF patients undergoing PCI, given its superior bleeding profile compared with ticagrelor or prasugrel. Triple antithrombotic therapy (OAC + aspirin + clopidogrel) should be limited to the periprocedural period (typically ≤1 week post-PCI), followed by dual therapy (OAC + clopidogrel) for 6–12 months based on individual ischemic versus hemorrhagic risk balance, with long-term oral anticoagulant monotherapy considered thereafter [42].

### 12.2. Elderly, Frail, and HBR Patients

Advanced age is another well-established major predictor of bleeding, as recognized across multiple validated risk scores and guideline frameworks [3,15,42,43]. The HOST-REDUCE-POLYTECH-ACS trial indicated that a reduction of prasugrel dosage to 5 mg within one month in elderly/low body weight patients led to a 30% reduction in NACE (HR: 0.70; *p* = 0.012) [47]. On the other hand, the use of DAPT in patients with severe renal dysfunction or life expectancies restricted due to cancer is not recommended [4]. Both the 2017 ESC DAPT update and the 2018 ESC/EACTS revascularization guidelines acknowledge advanced age as a significant modifier of the DAPT benefit–risk balance, supporting abbreviated DAPT durations in elderly patients where hemorrhagic risk predominates, and recommending dose reduction strategies, such as prasugrel 5 mg, as an alternative to complete drug discontinuation in order to preserve a degree of ischemic protection while limiting cumulative bleeding exposure [42,43].

### 12.3. Diabetes, CKD, and High Ischemic Risk Groups

Diabetes mellitus and CKD are strong and well-validated predictors of the clopidogrel non-responder phenotype, recognized by the 2024 International Consensus Statement [15] and ESC guidelines [16,17]. The ABCD-GENE score combines these factors; a score > 10 is associated with a 48% increase in MACE and a 54% increase in death, justifying the escalation to potent P2Y12 inhibitors in these cohorts [25].

### 12.4. Ethnic and Sex-Based Differences

The East Asian paradox is an issue whereby patients belonging to the East Asian ethnic group have a higher risk of bleeding when on potent P2Y12 inhibitors while having a low ischemic threshold. As such, a specific PRU value cutoff point needs to be employed [84]. In addition, a recent single-author preprint study that has not yet undergone peer review (Iftikhar) reported that Arab ethnicity was associated with a greater risk of ischemic events compared with non-Arabs (aHR = 1.54) [19]; however, this finding requires confirmation in peer-reviewed, prospectively validated cohorts before clinical application.

Regarding sex, female sex is significantly associated with higher 30-day event rates in post-CABG populations [85]. Women tend to have higher baseline platelet reactivity and greater platelet aggregability than men [86], which may partly explain observed sex differences in both ischemic and bleeding outcomes after PCI. Pharmacodynamic studies have demonstrated lower on-treatment platelet inhibition with clopidogrel in women, which some investigators have attributed to hormonal modulation of *CYP2C19* activity [87], though this hypothesis remains incompletely established. Furthermore, female patients were systematically underrepresented in foundational DAPT trials such as TRITON-TIMI 38 [88] and PLATO (A Comparison of Ticagrelor (AZD6140) and Clopidogrel in Patients With Acute Coronary Syndrome) [89], with approximately 26% and 28% female enrollment, limiting the generalizability of primary outcomes to women. Recent meta-analysis on 20 trials including 71,272 patients suggests that sex may influence the effectiveness of antiplatelet de-escalation after PCI. DAPT discontinuation reduced MACE in women (HR 0.86, 95% CI 0.75–0.98) but not in men (HR 1.04, 95% CI 0.93–1.16), while major bleeding was reduced in men (HR 0.60, 95% CI 0.44–0.82) but not in women (HR 1.04, 95% CI 0.76–1.43), compared to standard DAPT [90].

### 12.5. Urgent Surgery and Bridging Strategies

For patients requiring urgent surgery, short-acting parenteral agents like cangrelor provide a bridging strategy that allows for rapid platelet recovery upon cessation [8]. There are other agents, such as indobufen, which have a shorter half-life as compared to aspirin, reducing perioperative bleeding risk while maintaining antithrombotic protection [4]. The perioperative management of DAPT is critically time-dependent, and current guidelines provide a scenario-based framework for decision-making (Table 3).

The 2024 AHA/ACC Perioperative Guideline recommends delaying elective non-cardiac surgeries by 12 or more months after DES-PCI placed for ACS and 6 or more months after DES-PCI for chronic coronary disease [91]. In time-sensitive cases, a minimum 3-month period is considered for non-cardiac surgery. Elective non-cardiac surgery requiring interruption of any antiplatelet agent within 30 days of PCI is classified as potentially harmful [91].

*Preoperative P2Y12 Discontinuation:* Standard washout intervals are clopidogrel 5–7 days, ticagrelor 3–5 days, and prasugrel 7–10 days before non-cardiac surgery [91]. The Society of Thoracic Surgeons (STS) and Society of Cardiovascular Anesthesiologists (SCA) 2021 guidelines support point-of-care PFT to individualize surgical timing rather than relying solely on fixed washout periods. The American College of Chest Physicians (ACCP) 2022 guidelines recommend against routine PFT use while acknowledging potential benefits in select scenarios [92,93].

*Perioperative Aspirin Management:* Aspirin (75–100 mg) should be continued perioperatively in all patients with prior PCI.

*Intravenous Bridging (<6 Months Post-PCI):* In patients with high thrombotic risk undergoing nondeferrable non-cardiac surgery within 6 months of DES implantation or 30 days of bare metal stent implantation, intravenous bridging with cangrelor may be considered, starting 48 h after oral P2Y12 discontinuation and stopping 1–2 h before surgery [91,94]. GPIIb/IIIa inhibitors are an alternative when cangrelor is unavailable, stopping 6–8 h before surgery, though no validated bridging dose exists [94].

*Postoperative Resumption:* P2Y12 inhibitors should be reloaded with a loading dose within 24–72 h postoperatively when hemostasis is secured, as most ischemic events occur in the postoperative window [91,94].

## 13. Emerging Technologies and Future Directions

### 13.1. AI-Based Risk Prediction Models and Digital Health Tools

AI is driving a paradigm shift from static risk scores to dynamic, high-dimensional predictive frameworks. A recent preprint study, not yet peer-reviewed, reported that a gradient-boosting machine learning model demonstrated improved discrimination for ischemic events compared with traditional scoring tools such as PRECISE-DAPT [19]. These models continuously integrate 42 clinical variables, capturing non-linear interactions that static models miss. However, these findings have not been externally validated or prospectively tested in a randomized trial, and caution is warranted in interpreting single, non-peer-reviewed model performance figures as evidence of clinical superiority. In addition, several critical limitations of AI in this context must be acknowledged. First, most published models lack adequate clinical interpretability; while SHAP values provide feature-level explanations, explainable AI (XAI) frameworks remain insufficiently mature for DAPT decision support at the bedside. Second, integration into electronic health record workflows faces significant barriers, including heterogeneous data formats, missing variables, alert fatigue, and lack of interoperability standards. Third, no AI-based DAPT model has received regulatory clearance from the U.S. Food and Drug Administration or the European CE mark, precluding recommendation for routine clinical use. Fourth, existing models were trained on retrospective, single-center or population-specific cohorts, limiting generalizability. Prospective, multicenter, randomized trials comparing AI-guided versus guideline-based DAPT are urgently needed to establish clinical validity and safety.

The future of personalization relies on the seamless integration of predictive models into electronic health records to provide real-time clinical decision support. Mobile applications like those used in the MedApp-CHD (MEDication reminder APPs to improve medication adherence in Coronary Heart Disease) study are emerging as vital tools to improve medication adherence, a critical modifiable risk factor [95]. Further, integrating wearable-derived data, such as continuous heart rate and physical activity levels, into AI models offers the potential for proactive personalization, detecting physiological shifts before clinical events occur [96].

### 13.2. Multi-Omics and Precision Pharmacology

Precision therapy is evolving toward a multi-omics approach, combining genomics (*CYP2C19* status) with proteomics and metabolomics to explain the 85% of clopidogrel response variability not accounted for by single-gene testing [15,97]. This allows for deep phenotyping, identifying biological signatures of thrombotic vulnerability [1].

### 13.3. Novel Antiplatelet Agents

Emerging pharmacological options include indobufen, a reversible COX-1 inhibitor with significantly lower gastrointestinal risk than aspirin, and Factor XI inhibitors, which aim to prevent pathological thrombosis while preserving natural hemostasis [4,8].

## 14. Gaps in Evidence

The majority of previous studies have considered patient outcome measures based on a short surveillance period (approximately from 180 days to 1 year), making it difficult to reliably assess the long-term effects of personalized treatment algorithms on disease prognosis. An obvious gap is a lack of a sufficient observation period (5 years or more) for assessing the sustainability of computational approaches. Additionally, there is an absence of comparative analysis comparing various de-escalation methods, including aspirin-free P2Y12 inhibitor treatment versus PFT/genotype-based drug switch. Critically, there are no head-to-head randomized controlled trials directly comparing PFT-guided versus genotype-guided de-escalation strategies. This is a fundamental unresolved question with direct clinical implications: while both approaches have demonstrated safety in individual trials, it remains unknown whether genetic testing alone (identifying metabolizer status) provides equivalent or superior guidance to PFT, measuring the functional platelet inhibitory response, which integrates genetic, pharmacokinetic, and environmental factors.

Many personalized models utilize a narrow panel of single-gene tests; however, it has been shown that multiple genes are involved in antiplatelet activity regulation, including *P2RY12*, *CYP3A4*, and *ABCB1*, in addition to *CYP2C19*. Also, insufficient utilization of multi-gene panels and functional platelet tests limits their prediction accuracy. Moreover, there is no use of important predictive features, such as frailty indices, long-term adherence measures, and platelet activity changes.

Technically speaking, there are many issues surrounding the quality and consistency of electronic health record data, and these form major barriers to developing and validating reliable AI-based predictive algorithms.

Lastly, population heterogeneity is still underexplored. The ethnic disparities associated with a higher risk of bleeding and lower threshold for ischemia, known as the East Asian paradox, and the increased risks seen in the Arab population underscore the need for internationally validated risk prediction models.

## 15. Future Trial Design

Research in the future needs to shift focus toward dynamic, privacy-centric models using multimodal data. Federating learning that would help develop robust models without compromising on data governance issues when performed in multinational registries will be critical. Future clinical decision support systems must have electronic health record connectivity in real time and explainable AI (XAI). However, current problem-solving approaches remain highly individualized and are still dependent on clinical variables, clinician judgment, and prior experience. In this context, AI could help reduce such variability, although its full potential is not yet realized. Precision can be improved by using multiple genes and physiological parameters obtained from wearables. Lastly, future clinical trials need to continue surveillance for at least five years.

## 16. Conclusions

In conclusion, the growing body of evidence supports a more individualized approach to DAPT following myocardial revascularization, moving beyond traditional uniform treatment strategies. Clinical risk scores, platelet function testing, and *CYP2C19* genotyping can help optimize the balance between ischemic and bleeding risks in selected patients, while emerging AI-based tools may further enhance risk prediction and therapeutic decision-making. Although important knowledge gaps remain and many precision-guided strategies require further validation, integrating clinical, genetic, and phenotypic information offers a promising framework for improving antithrombotic care and advancing precision cardiovascular medicine.

## Figures and Tables

**Table 1:** Clinical risk stratification scores.

Score	Number of Participants	Variables	Discrimination (c-Statistic)	Clinical Application
DAPT score	11,648 patients	8 (5 clinical + 3 procedural)	0.70	Identify candidates for extended DAPT (>12 months)
PRECISE-DAPT	14,963 patients	5 clinical	0.73 (bleeding)	Identify HBR patients (score ≥ 25) for shortened DAPT
ARC-HBR	Consensus-based	13 criteria (major + minor)	N/A (semiquantitative)	Define HBR for clinical trials and practice
PARIS	8665 patients	6 clinical	0.65 (thrombosis), 0.64 (bleeding)	Dual assessment of thrombosis and bleeding risk
ABCD-GENE	3046 patients	5 (clinical + genetic)	0.71	Predict clopidogrel nonresponse; score > 10 = high MACE risk

DAPT, dual antiplatelet therapy; HBR, high bleeding risk; MACE, major adverse cardiovascular event.

**Table 2:** Studies of antiplatelet therapy after CABG.

Study	Participants	Intervention	Comparator	Primary Endpoint	Key Efficacy Result	Bleeding Result
DACAB (2018) [51]	500 (elective CABG—75% off-pump)	Ticagrelor + ASA × 1 yr; or ticagrelor alone × 1 yr	ASA alone × 1 yr	SVG patency at 1 yr	SVG patency: 88.7% (ticagrelor + ASA) vs. 82.8% (ticagrelor alone) vs. 76.5% (ASA alone); ticagrelor + ASA vs. ASA: *p* = 0.001	5 major bleeding episodes across all groups
Agrawal meta-analysis (2024) [48]	77,447 (CABG -mixed ACS/elective)	DAPT (any P2Y12 + ASA)	SAPT (ASA alone)	All-cause mortality, MACCE	↓ All-cause mortality (OR 0.65; 95% CI 0.50–0.86; *p* = 0.002); ↓ MACCE (OR 0.68; 95% CI 0.51–0.91; *p* = 0.01)	↑ Major bleeding (OR 1.30; 95% CI 1.08–1.56; *p* = 0.007)
Agrawal (DAPT-ticagrelor/prasugrel vs. DAPT-clopidogrel subgroup) (2024) [48]	Subset (CABG)	DAPT with ticagrelor/prasugrel	DAPT with clopidogrel	All-cause mortality	↓ All-cause mortality (OR 0.43; 95% CI 0.29–0.65; *p* ≤ 0.0001); ↓ CV mortality (OR 0.44; 95% CI 0.24–0.80)	No significant difference in bleeding
Agarwal meta-analysis (2018) [49]	11,135 (CABG)	DAPT	ASA alone	MACE, graft occlusion	↓ Graft occlusion (RR 0.79; 95% CI 0.63–0.98); ↓ all-cause mortality (RR 0.67; 95% CI 0.48–0.94)	No significant increase in major bleeding
Wang (2025) [52]	2424 (CABG)	Ticagrelor-based DAPT	Clopidogrel-based DAPT	Mortality, MACE	↓ Mortality and ↓ MACE with ticagrelor-based regimens	No significant difference in bleeding
POPular CABG (2020) [50]	499 (CABG with ≥1 SVG -31% ACS; 95% on-pump)	Ticagrelor + ASA × 1 yr	Placebo + ASA × 1 yr	SVG occlusion at 1 yr	No significant difference: 10.5% vs. 9.1% (OR 1.29; 95% CI 0.73–2.30; *p* = 0.38)	No significant difference
TACSI (2025) [57]	2201 (CABG for ACS -Nordic centers)	Ticagrelor + ASA × 1 yr	ASA alone × 1 yr	Death, MI, stroke, or repeat revascularization at 1 yr	No difference: 4.8% vs. 4.6% (HR 1.06; 95% CI 0.72–1.56; *p* = 0.77)	Major bleeding: 4.9% vs. 2.0% (HR 2.50; 95% CI 1.52–4.11)
Sandner IPD meta-analysis (2022) [58]	1316 (CABG with SVGs)	Ticagrelor + ASA	ASA alone	SVG failure per graft	↓ SVG failure: 11.2% vs. 20.0% (OR 0.51; 95% CI 0.35–0.74; *p* = 0.001)	↑ Clinically important bleeding (BARC 2/3/5) with ticagrelor DAPT
Bhatt review (2023) [53]	(CABG)	—	—	—	Highlights conflicting findings between DACAB, POPular CABG, and TARGET trials; no consensus on optimal DAPT duration	—
Bacha meta-analysis with TSA (2026) [59]	4208 (CABG)	Ticagrelor-based therapy	ASA alone	MACE, all-cause mortality, SVG failure	No difference in MACE (RR 1.05; *p* = 0.75) or mortality (RR 1.02; *p* = 0.93); ↓ SVG failure (RR 0.62; 95% CI 0.50–0.78; *p* = 0.0001)	No significant difference in major bleeding (RR 1.09; *p* = 0.73)
OPCAB personalized DAPT study (2022) [60]	1134 (OPCAB)	Personalized DAPT (*CYP2C19* genotyping + PFT-guided)	Standard DAPT (ASA + clopidogrel)	MACE	↓ MACE: 5.5% vs. 9.2% (HR 0.583; 95% CI 0.371–0.915; *p* = 0.019); ↓ MI: 1.7% vs. 4.2% (HR 0.407; *p* = 0.016)	No significant difference in TIMI major bleeding (5.3% vs. 6.0%; RR 0.883; *p* = 0.626)
Qu (2021) [61]	18,069 (CABG)	Clopidogrel + ASA	ASA alone	Composite (death, MI, stroke, revascularization) at 6 mo	↓ Composite: 2.9% vs. 4.2% (HR 0.65; 95% CI 0.55–0.77; *p* = 0.001)	No significant increase in major bleeding (HR 1.11; 95% CI 0.69–1.78)

ACS, acute coronary syndrome; ASA, aspirin; BARC, Bleeding Academic Research Consortium; CABG, coronary artery bypass grafting; CV, cardiovascular; DAPT, dual antiplatelet therapy; IPD, individual participant data; MACE, major adverse cardiac events; MACCE, major adverse cardiac and cerebrovascular events; MI, myocardial infarction; PFT, platelet function test; SAPT, single antiplatelet therapy; SVG, saphenous vein graft; TIMI, thrombolysis in myocardial infarction score; TSA, trial sequential analysis, ↑ Increase, ↓ Decrease.

**Table 3:** Perioperative antiplatelet management strategies.

Clinical Scenario	Recommended Surgical Delay	P2Y12 Discontinuation Timing	ASA Management	Bridging Strategy	Postoperative Resumption
Elective NCS after DES-PCI for ACS	≥12 months (Class I, LOE B-NR)	Clopidogrel: 5–7 days; Ticagrelor: 3–5 days; Prasugrel: 7–10 days	Continue ASA 75–100 mg (Class I, LOE B-R)	Not routinely required	Reload P2Y12 with loading dose within 24–72 h when hemostasis secured
Elective NCS after DES-PCI for CCD	≥6 months (Class IIa, LOE B-NR)	Clopidogrel: 5–7 days; Ticagrelor: 3–5 days; Prasugrel: 7–10 days	Continue ASA 75–100 mg (Class I, LOE B-R)	Not routinely required	Reload P2Y12 with loading dose within 24–72 h when hemostasis secured
Time-sensitive NCS after DES-PCI	≥3 months may be considered if risk of delay outweighs MACE risk (Class IIb, LOE B-NR)	Clopidogrel: 5–7 days; Ticagrelor: 3–5 days; Prasugrel: 7–10 days	Continue ASA 75–100 mg	Consider IV bridging (see below)	Reload P2Y12 with loading dose within 24–72 h when hemostasis secured
NCS within 30 days of BMS- or DES-PCI	Elective NCS: potentially harmful (Class III: Harm, LOE B-NR)	Continue DAPT if feasible; if must interrupt, minimize duration	Continue DAPT if feasible	IV bridging strongly considered	Reload P2Y12 as soon as possible
NCS after PCI (ESC perspective) [16,17]	≥1 month irrespective of stent type (Class IIa, LOE B); ≥6 months if recent MI or high ischemic risk (Class IIb, LOE C)	Ticagrelor: ≥3 days; Clopidogrel: ≥5 days; Prasugrel: ≥7 days (Class IIa, LOE B)	Continue ASA (Class I, LOE B); resume P2Y12 as soon as possible postoperatively	IV antiplatelet bridging may be considered if surgery within 1 month of stent (Class IIb, LOE C)	Resume P2Y12 as soon as possible postoperatively (Class I, LOE B)
Elective CABG	Ticagrelor: minimum 3 days; Clopidogrel: 5 days; Prasugrel: 7 days (Class II, LOE B-NR)	Per above	Continue ASA through surgery (suggested over interruption)	Not routinely indicated	Resume ASA or P2Y12 within 24 h after surgery
CABG—role of PFT	POC PFT can guide timing of surgery (Class IIa, LOE B-R)	Individualized based on PFT results	Continue ASA	Not applicable	Resume within 24 h
Elective NCS (general)	—	Clopidogrel: 5–7 days; Ticagrelor: 3–5 days; Prasugrel: 7–10 days	Continue ASA if possible; if must stop, ≤7 days before surgery	Not addressed	Resume within 24 h
NCS—role of PFT	—	—	—	—	—
IV bridging with cangrelor (high thrombotic risk, 6 months post-DES or 30 days post-BMS)	Surgery nondeferrable	Start cangrelor 0.75 μg/kg/min (bridging dose) 48 h after oral P2Y12 discontinuation (or 2–3 days after clopidogrel/ticagrelor; 3–4 days after prasugrel)	Continue ASA throughout	Cangrelor: stop 1–2 h before surgery; platelet function recovers within 60 min	Reload oral P2Y12 (preferably clopidogrel 300–600 mg) within 6 h postoperatively if hemostasis permits
IV bridging with GPIIb/IIIa inhibitors (when cangrelor unavailable)	Surgery nondeferrable	Per standard washout	Continue ASA throughout	Eptifibatide 2.0 μg/kg/min or tirofiban 0.1 μg/kg/min; stop 6–8 h before surgery; no validated bridging dose exists	Reload oral P2Y12 within 6 h postoperatively if hemostasis permits

ACS, acute coronary syndrome; ASA, aspirin; BMS, bare metal stent; CABG, coronary artery bypass grafting; CCD, chronic coronary disease; DAPT, dual antiplatelet therapy; DES, drug-eluting stent; ESC, European Society of Cardiology; IV, intravenous; LOE, level of evidence; MACE, major adverse cardiac events; MI, myocardial infarction; NCS, non-cardiac surgery; PCI, percutaneous coronary intervention; PFT, platelet function test; POC, point of care.

**Table 4:** Studies of DAPT duration and de-escalation after PCI.

Study	Participants	Intervention	Comparator	Primary Endpoint	Key Efficacy Result	Bleeding Result
**DAPT duration/P2Y12 monotherapy trials**						
STOPDAPT-2 (2019) [69]	3045 (PCI with DES—38% ACS)	1-mo DAPT → clopidogrel mono	12-mo DAPT (ASA + clopidogrel)	NACE at 1 year	Superior: 2.36% vs. 3.70% (HR 0.64; *p* = 0.04)	TIMI major/minor: HR 0.46; 95% CI 0.23–0.94
MASTER DAPT (2021) [70]	4579 (HBR patients with DES)	1-month DAPT → SAPT	≥3-month DAPT (standard)	NACE + MACE co-primary at 1 year	Non-inferior for both NACE and MACE	Substantially reduced major bleeding
SMART-DATE (2018) [71]	2712 (ACS with DES—38% STEMI)	6-month DAPT	≥12-month DAPT	MACCE at 18 months	Non-inferior (*p* = 0.03 for NI)	Numerically lower (NS)
REDUCE-ACS (2019) [72]	1496 (ACS with DES—47% STEMI)	3-month DAPT	12-month DAPT	NACE at 1 year	Non-inferior (*p* = 0.001 for NI)	Numerically lower (NS)
TWILIGHT (2019) [73]	7119 (high-risk PCI—64% ACS)	3-month DAPT → ticagrelor mono	12-month DAPT (ASA + ticagrelor)	BARC 2/3/5 bleeding at 1 year	Death/MI/stroke: 3.9% vs. 3.9% (non-inferior)	BARC 2/3/5: 4.0% vs. 7.1% (HR 0.56; *p* = 0.001)
ULTIMATE-DAPT (2024) [74]	3400 (ACS with DES)	1- month DAPT → ticagrelor mono	12-month DAPT (ASA + ticagrelor)	Clinically relevant bleeding at 1 year	MACCE: no significant difference	Major bleeding: 2.1%—significantly reduced
**De-Escalation Trials**						
TOPIC (2017) [45]	646 (ACS-PCI, event-free at 1 month)	Unguided switch to clopidogrel + ASA at 1 month	Standard DAPT (potent P2Y12 + ASA)	NACE at 1 year	HR 0.48; 95% CI 0.34–0.68; *p* = 0.01 (52% ↓ NACE)	BARC ≥ 2: HR 0.30; 95% CI 0.18–0.50
TROPICAL-ACS (2017) [46]	2610 (ACS-PCI)	PFT-guided de-escalation to clopidogrel at 14 days	Standard prasugrel × 12 months	NACE at 1 year	Non-inferior (HR 0.81; *p* = 0.0004 for NI)	BARC ≥ 2: HR 0.82; 95% CI 0.59–1.13 (NS)
HOST-REDUCE-POLYTECH-ACS (2020) [47]	2338 (ACS-PCI)	Prasugrel 5 mg at 1 month (dose de-escalation)	Prasugrel 10 mg × 12 months	NACE at 1 year	Non-inferior → superior (HR 0.70; 95% CI 0.52–0.92; *p* = 0.012)	BARC ≥ 2: HR 0.48; 95% CI 0.32–0.73
TALOS-AMI (2021) [44]	2697 (AMI-PCI, event-free at 1 month)	Unguided switch to clopidogrel + ASA at 1 month	Ticagrelor + ASA × 12 months	NACE at 1 year	Non-inferior → superior (HR 0.55; 95% CI 0.40–0.76; *p* = 0.0001)	BARC 2/3/5: 3.0% vs. 5.6% (HR 0.52; *p* = 0.0012)
**Guided- Therapy Trials**						
PATH-PCI [40]	CCS after PCI	PFT-guided antiplatelet management	Standard therapy	NACE	32% ↓ NACE (HR 0.68; 95% CI 0.49–0.95)	—
PHARMCLO (2018) [41]	888 (ACS)	Genotype-guided antiplatelet selection	Standard therapy	NACE	42% ↓ NACE (HR 0.58)	—
GRAVITAS (2011) [30]	2214 (PCI with HTPR -PRU ≥ 230)	High-dose clopidogrel (150 mg)	Standard-dose clopidogrel (75 mg)	Death, MI, or stent thrombosis at 6-month	No significant difference: 2.3% vs. 2.3% (HR 1.01; *p* = 0.98)	No significant difference

ACS, acute coronary syndrome; AMI, acute myocardial infarction; ASA, aspirin; BARC, Bleeding Academic Research Consortium; CCS, chronic coronary syndrome; DAPT, dual antiplatelet therapy; DES, drug-eluting stent; HBR, high bleeding risk; HTPR, high on-treatment platelet reactivity; MACE, major adverse cardiac events; MACCE, major adverse cardiac and cerebrovascular events; MI, myocardial infarction; NACE, net adverse clinical events; NI, non-inferiority; NS, non-standard; PCI, percutaneous coronary intervention; PFT, platelet function test; PRU, platelet reactivity unit; SAPT, single antiplatelet therapy; STEMI, ST-segment elevation myocardial infarction; TIMI, thrombolysis in myocardial infarction score.

## Data Availability

Data sharing is not applicable to this article, as no datasets were generated or analyzed during this work.

## Abbreviations

The following abbreviations are used in this manuscript:

ACC	American College of Cardiology
ACS	Acute coronary syndrome
ADP	Adenosine diphosphate
AHA	American Heart Association
AI	Artificial intelligence
CABG	Coronary artery bypass grafting
CAD	Coronary artery disease
CKD	Chronic kidney disease
DAPT	Dual antiplatelet therapy
DES	Drug-eluting stent
ESC	European Society of Cardiology
HBR	High bleeding risk
HTPR	High on-treatment platelet reactivity
LoF	Loss of function
MACE	Major adverse cardiovascular events
MI	Myocardial infarction
NACE	Net adverse clinical events
PCI	Percutaneous coronary intervention
PFT	Platelet function testing
PRU	Platelet reactivity units
SCAI	Society for Cardiovascular Angiography and Interventions
SVG	Saphenous vein graft
